# 1-[2-(4-Nitrophenyl)-5-(5-phenyl-1,2-oxazol-3-yl)-1,2,3,4-tetrahydroquinolin-4-yl]pyrrolidin-2-one

**DOI:** 10.1107/S1600536810054048

**Published:** 2011-01-08

**Authors:** Margarita Gutierrez, Luis Astudillo, Luisa Quesada, Iván Brito, Matías López-Rodríguez

**Affiliations:** a Instituto de Quimica de Recursos Naturales, Universidad de Talca, Casilla 747, Talca, Chile; b Departamento de Química, Facultad de Ciencias Básicas, Universidad de Antofagasta, Casilla 170, Antofagasta - Chile; c Instituto de Bio-Orgánica ’Antonio González’, Universidad de La Laguna, Astrofísico Francisco Sánchez N°2, La Laguna, Tenerife, Spain.

## Abstract

The title compound, (I) C_28_H_24_N_4_O_4_, is the *trans* diastereo­isomer of the compound 1-[2-(4-nitro­phen­yl)-6-(5-phenyl-3-isoxazol­yl)-1,2,3,4-tetra­hydro-4-quinolin­yl]-2-pyrrolidinone monohydrate, (II) [Gutierrez *et al.* (2011 ▶). *Acta Cryst.* E**67**, o175–o176]. The most obvious differences between the diastereo­isomers are the dihedral angles between the isoxazole ring and the benzene and phenyl rings [47.0 (2); 56.4 (2) and 33.3 (2); 11.0 (2)°, respectively, for (II) 75.4 (2) and 5.8 (3), respectively, for (I)]. In the crystal of (I), the mol­ecules are linked by N—H⋯O inter­actions into a chain along [001] with graph-set notation *C*(8).

## Related literature

For details of nitro­gen-containing heterocyclic compounds, see: Sankaran *et al.* (2010 ▶) and for their pharmacological activity, see: Shi *et al.* (2008 ▶); Lunniss *et al.* (2009 ▶); He *et al.* (2005 ▶); Eswaran *et al.* (2010 ▶). For reactions of isoxazoles, see: Taldone *et al.* (2008 ▶); Narlawar *et al.* (2008 ▶); Velaparthi *et al.* (2008 ▶); Rizzi *et al.* (2008 ▶); Lautens & Roy (2000 ▶); Broggini *et al.* (2005 ▶); Kotera *et al.* (1970 ▶). For applications of compounds possessing the quinoline system as drugs and pharmaceuticals, see: Kalita *et al.* (2006 ▶). For syntheses of quinolines, see: Kouznetsov *et al.* (2005 ▶). For the *trans* diastereoisomer of the title compound, see: Gutierrez *et al.* (2011 ▶). For graph-set motifs see: Bernstein *et al.* (1995 ▶) and for puckering parameters, see: Cremer & Pople (1975 ▶)
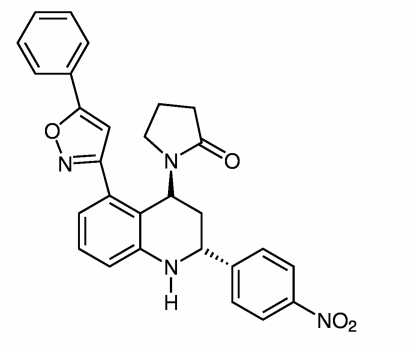


## Experimental

### 

#### Crystal data

C_28_H_24_N_4_O_4_
*M* *_r_* = 480.51
Hexagonal, 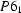

*a* = 20.753 (3) Å
*c* = 10.446 (2) Å
*V* = 3896.2 (11) Å^3^
*Z* = 6
Mo *K*α radiation
μ = 0.08 mm^−1^
*T* = 293 K
0.20 × 0.20 × 0.18 mm

#### Data collection

Nonius KappaCCD area-detector diffractometer
5951 measured reflections
3144 independent reflections
2646 reflections with *I* > 2σ(*I*)
*R*_int_ = 0.026

#### Refinement

*R*[*F* ^2^ > 2σ(*F* ^2^)] = 0.075
*wR*(*F* ^2^) = 0.162
*S* = 1.22
3144 reflections
329 parameters
1 restraint
H atoms treated by a mixture of independent and constrained refinement
Δρ_max_ = 0.32 e Å^−3^
Δρ_min_ = −0.24 e Å^−3^

### 

Data collection: *COLLECT* (Nonius, 2000 ▶); cell refinement: *DENZO-SMN* (Otwinowski & Minor, 1997 ▶); data reduction: *DENZO-SMN*; program(s) used to solve structure: *SIR97* (Altomare *et al.*, 1999 ▶); program(s) used to refine structure: *SHELXL97* (Sheldrick, 2008 ▶); molecular graphics: *ORTEP-3 for Windows* (Farrugia, 1997 ▶) and *PLATON* (Spek, 2009 ▶); software used to prepare material for publication: *WinGX* (Farrugia, 1999 ▶).

## Supplementary Material

Crystal structure: contains datablocks I, global. DOI: 10.1107/S1600536810054048/sj5084sup1.cif

Structure factors: contains datablocks I. DOI: 10.1107/S1600536810054048/sj5084Isup2.hkl

Additional supplementary materials:  crystallographic information; 3D view; checkCIF report

## Figures and Tables

**Table 1:** Hydrogen-bond geometry (Å, °)

*D*—H⋯*A*	*D*—H	H⋯*A*	*D*⋯*A*	*D*—H⋯*A*
N2—H2*N*⋯O4^i^	0.87 (3)	1.99 (3)	2.859 (6)	180 (5)

Symmetry code: (i) 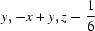.

**Fig. 1.**
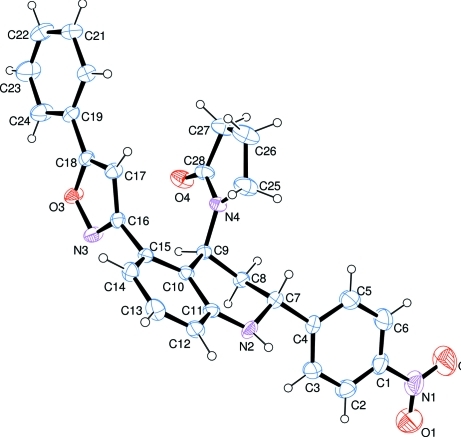
The structure of (I), showing the atom-numbering scheme. Displacement ellipsoids are plotted at the 30% probability level.

**Fig. 2.**
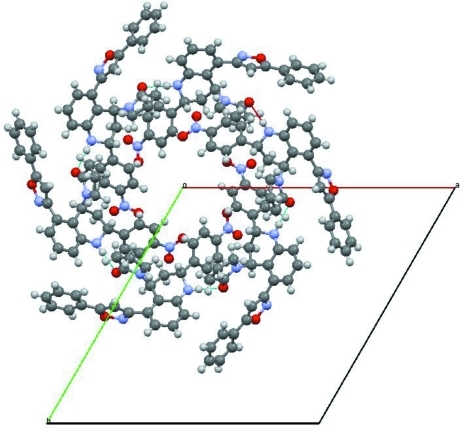
A view of (I), showing the mono-dimensional framework constructed via N—H···O hydrogen bonds. Hydrogen bonds are depicted as dashed lines [symmetry-code:(i) y, -x + y, z - 1/6.]

**Fig. 3.**
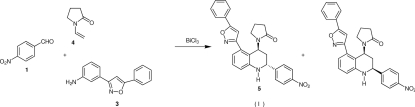
The preparation of the title compound.

## supplementary crystallographic information

### Comment

Nitrogen containing heterocycles are indispensable structural units for medicinal chemists (Sankaran *et al.*, 2010). Compounds possessing the quinoline system have wide applications as drugs and pharmaceuticals and also occur as the structural framework in some natural products (Kalita *et al.*, 2006). They also have several pharmacological activities as anti-breast cancer (Shi *et al.*, 2008), selective PDE4 inhibition (Lunniss *et al.*, 2009), immuno modulatory (He *et al.*, 2005), and antimycobacterial agents (Eswaran *et al.*, 2010), among others.

Quinoline and its derivatives represent a major class of heterocycles, and a number of preparations have been known since the late 1800's. The quinoline skeleton is often used for the design of many synthetic compounds with diverse pharmacological properties. Several syntheses of quinolines are known, but due to their importance, the development of new synthetic approaches remains an active research area (Kouznetsov *et al.*, 2005).

The isoxazoles form a relevant group of biologically active compounds with a wide range of applications, including Hsp90 super chaperone complex inhibitors (Taldone *et al.*, 2008), tau aggregation inhibitors for treatment of Alzheimer's disease (Narlawar *et al.*, 2008), *mycobacterium tuberculosis* pantothenate synthetase inhibitors (Velaparthi *et al.*, 2008) and neuronal nicotinic acetylcholine receptor agonists (Rizzi *et al.*, 2008).

A considerable number of methods to synthesize substituted isoxazoles have been published including approaches based on intramolecular cycloadditions, condensations, and intramolecular cyclizations of amino acids. These methods sometime suffer in their versatility, convenience and yield (Lautens & Roy, 2000). The isoxazole ring can be synthetized by 1, 3-dipolar cyclo-addition reactions between a nitrile oxide and an alkyne, that reaction may be catalyzed by copper(II). Cycloaddition reactions are among the most useful reactions in synthetic and mechanistic organic chemistry (Broggini *et al.*, 2005).

Isoxazoles have a rich chemistry because of their easy reductive cleavage and susceptibility to ring transformations (Kotera *et al.*, 1970). Depending on the substitution patterns, isoxazoles can be used as reagents for the imino-Diels-Alder condensation between anilines, aldehydes and electron-rich alkenes to generate tetrahydroquinolines with different selected substitution patterns.

Due to these facts, the combination of the two heterocyclic rings into a new chemical entity is of interest, as no examples are known in the chemical literature to date.

Many molecules widely used today consist of fusions of rings; an example is the case of penicillins, where incorporation of an isoxazole ring led to the formation of stable derivatives which catalyzed the degradation of gastric acid levels (flucloxacillin and cloxacillin).

We report here the crystal structure of a novel synthetic derivative *cis* quinoline-isoxazole prepared by imino Diels-Alder cyclo-addition, Scheme 1.

The structure of the title compound, (I) C_28_H_24_N_4_O_4_, has hexagonal (*P*6_1_) symmetry and is the *trans* diastereoisomer of the compound 1-[2-(4-nitrophenyl)-6-(5-phenyl-3-isoxazolyl)-1,2,3,4-tetrahydro-4-\ quinolinyl]-2-pyrrolidinone-dihydrate, (II) (Gutierrez *et al.*, 2011), so the pyrrolidinone fragment is *trans* oriented respect to 4-nitrophenyl fragment [C7—C8—C9—N4 torsion angle 85.59 (4)°; -175.(2) (4)° for (II) (mean)]. The most obvious differences between both diastereoisomers are the torsion angles between the isoxazole ring and the benzene and phenyl rings [47.0 (2); 56.4 (2) and 33.3 (2); 11.0 (2)° for (II) 75.4 (2) and 5.8 (3) for (I)]. In both diastereoisomers the six-membered heterocyclic ring has a half-boat conformation (Q_T_= 0.477 (5) Å, θ = 47.7 (5)° φ= 76.3 (7)°), Cremer & Pople, 1975. In the crystal, molecules are linked by N— H··· O interactions into chains with graph-set notation C(8) along [001], Fig. 2, Bernstein *et al.*, 1995.

### Experimental

A mixture of 3-(3-aminophenyl)-5-phenylisoxazole (2.8 mmol) 3 and 4-nitrobenzaldehyde (3.4 mmol) 1 in anhydrous CH_3_CN (15 mL) was stirred at room temperature for 30 min. BiCl_3_ (2.0 mmol) was added. Over a period of 20 min, a solution the *N*-vinyl.2-pyrrolidone (NVP) (5.5 mmol) 4 in CH_3_CN (10 ml) was added dropwise. The resulting mixture was stirred for 10–14 h. After completion of the reaction as indicated by TLC, the reaction mixture was diluted with water (30 mL) and extracted with ethyl acetate (3×15 mL). The organic layer was separated and dried (Na_2_SO_4_),concentrated in vacuum and the resulting product was purified by column chromatography (silica gel) using PE and EtOAc mixtures. Obtained for derivatives *trans* and *cis* Quinoline-Isoxazole 5 and (I), see Figure 3. Solid crystalline mp 130 - 132 °C; RMN-^1^H(CDCl_3_), 400 MHz, δ): 8.24 (2*H*, d, *J* = 8.0); 7.81 (2*H*, d, *J*= 8.0); 7.60 (1*H*, d, *J* = 8.0); 7.27(2*H*, dd, *J* = 8.0 and 4.0); 7.17 (1*H*,t, *J* = 8.0); 6.97 (1*H*, d, *J* = 8.0); 6.89 (2*H*, d, *J* = 8.0); 6.78 (1*H*, d, *J* = 8.0); 6.62 (1*H*, s); 5.35 (1*H*, s); 4.59 (1*H*, dd, *J* = 12.0 and 1.0); 4.41 (1*H*, br. s);2.39 (2*H*, m); 1.97 (2*H*, m); 1.62 (2*H*, s). RMN-^13^H (CDCl_3_),400 MHz, δ): 174.68, 169.95, 162.48, 150.61, 147.65, 145.15, 130.87, 129.31, 128.98, 127.47,127.27, 125.95, 125.04, 124.61, 119.61, 116.32, 115.27, 99.89, 52.54, 47.51,46.22, 37.37, 31.36, 18.56. MS *m/z* (EI): 480. Anal. Calcd. for C_28_H_24_N_4_O_4_: C, 69.99; H,5.03;N, 11.66. Found: C, 69.89; H, 5.01; N, 11.77.

### Refinement

The SQUEEZE function of *PLATON* (Spek, 2009) was used to eliminate the contribution of electron density in the solvent region from the intensity data, and a solvent-free model was employed for the final refinement. The volume which is accessible for potential solvent molecules was calculated to be 452.0 Å^3^ and the total electron count per cell was calculated to be 15. Note that the calculated density, the F(000) value, the molecular weight and the formula are given without taking into account the results obtained with the SQUEEZE option in *PLATON* (Spek, 2009). Therefore, the solvent-free model and intensity data were used for the final results reported here.

The absolute configuration of the two stereogenic centres could not be established by the Flack parameter (Flack, 1983) and Friedel opposites were merged.

The position of the N2 H atom was refined freely with isotropic displacement parameters. All other H atoms were placed in geometrically idealized positions (C—H = 0.93–0.97 Å) and constrained to ride on their parent atoms with *U*_iso_(H) = 1.2*U*_eq_(C).

### Crystal data

**Table:**

C_28_H_24_N_4_O_4_	*D*_x_ = 1.229 Mg m^−^^3^
*M**_r_* = 480.51	Mo *K*α radiation, λ = 0.71073 Å
Hexagonal, *P*6_1_	Cell parameters from 2728 reflections
Hall symbol: P 61	θ = 2.8–27.5°
*a* = 20.753 (3) Å	µ = 0.08 mm^−^^1^
*c* = 10.446 (2) Å	*T* = 293 K
*V* = 3896.2 (11) Å^3^	Prismatic, orange
*Z* = 6	0.20 × 0.20 × 0.18 mm
*F*(000) = 1512	

### Data collection

**Table:**

Nonius KappaCCD area-detector diffractometer	2646 reflections with *I* > 2σ(*I*)
Radiation source: fine-focus sealed tube	*R*_int_ = 0.026
graphite	θ_max_ = 27.5°, θ_min_ = 2.8°
φ scans, and ω scans with κ offsets	*h* = −22→0
5951 measured reflections	*k* = 0→26
3144 independent reflections	*l* = −13→13

### Refinement

**Table:**

Refinement on *F*^2^	Primary atom site location: structure-invariant direct methods
Least-squares matrix: full	Secondary atom site location: difference Fourier map
*R*[*F*^2^ > 2σ(*F*^2^)] = 0.075	Hydrogen site location: inferred from neighbouring sites
*wR*(*F*^2^) = 0.162	H atoms treated by a mixture of independent and constrained refinement
*S* = 1.22	*w* = 1/[σ^2^(*F*_o_^2^) + (0.0755*P*)^2^ + 0.8624*P*] where *P* = (*F*_o_^2^ + 2*F*_c_^2^)/3
3144 reflections	(Δ/σ)_max_ = 0.010
329 parameters	Δρ_max_ = 0.32 e Å^−^^3^
1 restraint	Δρ_min_ = −0.24 e Å^−^^3^

### Special details

**Table:**

Geometry. All s.u.'s (except the s.u. in the dihedral angle between two l.s. planes) are estimated using the full covariance matrix. The cell s.u.'s are taken into account individually in the estimation of s.u.'s in distances, angles and torsion angles; correlations between s.u.'s in cell parameters are only used when they are defined by crystal symmetry. An approximate (isotropic) treatment of cell s.u.'s is used for estimating s.u.'s involving l.s. planes.
Refinement. Refinement of *F*^2^ against ALL reflections. The weighted *R*-factor *wR* and goodness of fit *S* are based on *F*^2^, conventional *R*-factors *R* are based on *F*, with *F* set to zero for negative *F*^2^. The threshold expression of *F*^2^ > 2σ(*F*^2^) is used only for calculating *R*-factors(gt) *etc*. and is not relevant to the choice of reflections for refinement. *R*-factors based on *F*^2^ are statistically about twice as large as those based on *F*, and *R*- factors based on ALL data will be even larger.

### Fractional atomic coordinates and isotropic or equivalent isotropic displacement parameters (Å^2^)

**Table:**

	*x*	*y*	*z*	*U*_iso_*/*U*_eq_	
O1	0.2019 (3)	−0.0999 (3)	0.6005 (5)	0.1165 (18)	
O2	0.1082 (3)	−0.1080 (3)	0.5004 (7)	0.147 (3)	
O3	0.54683 (17)	0.55998 (14)	0.2252 (3)	0.0485 (7)	
O4	0.36115 (17)	0.42435 (19)	0.2789 (3)	0.0607 (8)	
N1	0.1743 (3)	−0.0784 (3)	0.5205 (5)	0.0880 (15)	
N2	0.43438 (18)	0.19821 (17)	0.2011 (4)	0.0456 (8)	
N3	0.5569 (2)	0.49764 (18)	0.2257 (4)	0.0492 (8)	
N4	0.36641 (16)	0.33963 (17)	0.1463 (3)	0.0388 (7)	
C1	0.2234 (3)	−0.0115 (2)	0.4445 (5)	0.0631 (12)	
C2	0.2982 (3)	0.0205 (3)	0.4559 (5)	0.0637 (12)	
H2	0.3186	−0.0002	0.5098	0.076*	
C3	0.3433 (2)	0.0833 (2)	0.3881 (5)	0.0597 (12)	
H3	0.3946	0.1050	0.3963	0.072*	
C4	0.3145 (2)	0.1154 (2)	0.3075 (4)	0.0489 (10)	
C5	0.2376 (3)	0.0806 (3)	0.2964 (7)	0.0855 (19)	
H5	0.2168	0.1006	0.2416	0.103*	
C6	0.1916 (3)	0.0171 (3)	0.3647 (7)	0.091 (2)	
H6	0.1401	−0.0057	0.3568	0.109*	
C7	0.3612 (2)	0.1867 (2)	0.2334 (5)	0.0471 (9)	
H7	0.3351	0.1839	0.1535	0.057*	
C8	0.3719 (2)	0.25488 (19)	0.3085 (4)	0.0404 (8)	
H8A	0.3237	0.2483	0.3310	0.048*	
H8B	0.3985	0.2591	0.3873	0.048*	
C9	0.41529 (18)	0.32633 (18)	0.2308 (4)	0.0337 (7)	
H9	0.4378	0.3677	0.2918	0.040*	
C10	0.47821 (18)	0.32842 (19)	0.1526 (4)	0.0335 (7)	
C11	0.4825 (2)	0.26296 (19)	0.1383 (4)	0.0379 (8)	
C12	0.5384 (2)	0.2653 (2)	0.0586 (4)	0.0451 (10)	
H12	0.5428	0.2231	0.0502	0.054*	
C13	0.5865 (2)	0.3290 (2)	−0.0069 (4)	0.0499 (10)	
H13	0.6217	0.3289	−0.0618	0.060*	
C14	0.5826 (2)	0.3935 (2)	0.0088 (4)	0.0439 (9)	
H14	0.6151	0.4365	−0.0357	0.053*	
C15	0.53053 (19)	0.39350 (19)	0.0906 (4)	0.0345 (8)	
C16	0.53036 (18)	0.46432 (19)	0.1164 (4)	0.0355 (8)	
C17	0.5044 (2)	0.5032 (2)	0.0404 (4)	0.0393 (8)	
H17	0.4844	0.4914	−0.0417	0.047*	
C18	0.51507 (19)	0.56076 (19)	0.1134 (4)	0.0374 (8)	
C19	0.4969 (2)	0.6206 (2)	0.0963 (4)	0.0401 (8)	
C20	0.4578 (2)	0.6207 (2)	−0.0102 (4)	0.0456 (9)	
H20	0.4425	0.5828	−0.0704	0.055*	
C21	0.4409 (2)	0.6770 (3)	−0.0284 (5)	0.0552 (11)	
H21	0.4141	0.6767	−0.0999	0.066*	
C22	0.4643 (3)	0.7329 (3)	0.0603 (6)	0.0655 (14)	
H22	0.4544	0.7714	0.0476	0.079*	
C23	0.5015 (4)	0.7325 (3)	0.1657 (6)	0.0790 (17)	
H23	0.5157	0.7701	0.2262	0.095*	
C24	0.5191 (3)	0.6767 (3)	0.1855 (5)	0.0671 (14)	
H24	0.5454	0.6773	0.2579	0.080*	
C25	0.3392 (3)	0.3060 (3)	0.0210 (5)	0.0674 (14)	
H25A	0.3053	0.2529	0.0290	0.081*	
H25B	0.3802	0.3141	−0.0341	0.081*	
C26	0.2998 (3)	0.3441 (4)	−0.0316 (6)	0.0799 (16)	
H26A	0.3271	0.3753	−0.1038	0.096*	
H26B	0.2503	0.3076	−0.0602	0.096*	
C27	0.2955 (3)	0.3900 (3)	0.0734 (5)	0.0704 (14)	
H27A	0.2446	0.3694	0.1028	0.085*	
H27B	0.3135	0.4407	0.0446	0.085*	
C28	0.3444 (2)	0.3884 (2)	0.1797 (4)	0.0474 (10)	
H2N	0.4315 (16)	0.1573 (18)	0.174 (3)	0.017 (8)*	

### Atomic displacement parameters (Å^2^)

**Table:**

	*U*^11^	*U*^22^	*U*^33^	*U*^12^	*U*^13^	*U*^23^
O1	0.113 (4)	0.106 (3)	0.110 (4)	0.039 (3)	0.008 (3)	0.058 (3)
O2	0.088 (3)	0.109 (4)	0.173 (6)	−0.005 (3)	−0.006 (4)	0.069 (4)
O3	0.0723 (18)	0.0384 (13)	0.0401 (15)	0.0317 (13)	−0.0135 (14)	−0.0066 (12)
O4	0.0657 (18)	0.086 (2)	0.0577 (19)	0.0587 (18)	−0.0052 (16)	−0.0198 (18)
N1	0.079 (3)	0.063 (3)	0.090 (4)	0.011 (2)	0.004 (3)	0.018 (3)
N2	0.0506 (18)	0.0267 (15)	0.062 (2)	0.0212 (14)	0.0040 (17)	−0.0034 (15)
N3	0.067 (2)	0.0429 (17)	0.0464 (19)	0.0341 (16)	−0.0109 (18)	−0.0027 (16)
N4	0.0363 (15)	0.0475 (16)	0.0394 (17)	0.0262 (14)	−0.0005 (14)	−0.0013 (14)
C1	0.066 (3)	0.040 (2)	0.064 (3)	0.012 (2)	0.001 (2)	0.007 (2)
C2	0.070 (3)	0.061 (3)	0.063 (3)	0.035 (2)	0.004 (3)	0.016 (2)
C3	0.052 (2)	0.057 (2)	0.068 (3)	0.026 (2)	−0.002 (2)	0.014 (2)
C4	0.046 (2)	0.0338 (18)	0.058 (3)	0.0127 (16)	−0.001 (2)	0.0012 (19)
C5	0.059 (3)	0.070 (3)	0.111 (5)	0.020 (2)	−0.008 (3)	0.038 (4)
C6	0.056 (3)	0.068 (3)	0.117 (5)	0.008 (3)	−0.013 (3)	0.029 (4)
C7	0.046 (2)	0.0363 (19)	0.055 (2)	0.0175 (16)	−0.0023 (19)	0.0047 (18)
C8	0.0396 (18)	0.0416 (19)	0.043 (2)	0.0225 (16)	0.0057 (17)	0.0045 (17)
C9	0.0337 (16)	0.0332 (17)	0.0379 (18)	0.0194 (14)	0.0005 (15)	−0.0010 (15)
C10	0.0337 (17)	0.0359 (17)	0.0354 (18)	0.0207 (15)	−0.0041 (15)	−0.0050 (15)
C11	0.0400 (18)	0.0380 (18)	0.044 (2)	0.0260 (16)	−0.0081 (17)	−0.0073 (17)
C12	0.042 (2)	0.044 (2)	0.059 (3)	0.0297 (18)	−0.007 (2)	−0.016 (2)
C13	0.0383 (19)	0.059 (2)	0.056 (3)	0.0276 (19)	0.008 (2)	−0.012 (2)
C14	0.0369 (18)	0.043 (2)	0.050 (2)	0.0181 (16)	0.0077 (18)	−0.0001 (19)
C15	0.0344 (17)	0.0341 (17)	0.0376 (18)	0.0192 (14)	−0.0063 (15)	−0.0017 (15)
C16	0.0317 (17)	0.0348 (17)	0.0365 (19)	0.0139 (14)	0.0064 (15)	0.0058 (15)
C17	0.0493 (19)	0.0404 (18)	0.0315 (19)	0.0249 (16)	0.0008 (16)	0.0012 (16)
C18	0.0385 (18)	0.0345 (18)	0.0376 (19)	0.0170 (15)	0.0034 (16)	0.0050 (15)
C19	0.047 (2)	0.0351 (18)	0.042 (2)	0.0236 (16)	0.0096 (17)	0.0055 (16)
C20	0.053 (2)	0.047 (2)	0.041 (2)	0.0287 (19)	0.0041 (19)	0.0014 (18)
C21	0.065 (3)	0.063 (3)	0.053 (2)	0.044 (2)	0.002 (2)	0.010 (2)
C22	0.093 (4)	0.055 (3)	0.070 (3)	0.053 (3)	0.012 (3)	0.010 (3)
C23	0.120 (5)	0.063 (3)	0.076 (4)	0.061 (3)	−0.020 (4)	−0.025 (3)
C24	0.101 (4)	0.057 (3)	0.059 (3)	0.051 (3)	−0.025 (3)	−0.017 (2)
C25	0.078 (3)	0.086 (3)	0.058 (3)	0.055 (3)	−0.024 (3)	−0.020 (3)
C26	0.083 (4)	0.119 (5)	0.059 (3)	0.067 (4)	−0.022 (3)	−0.011 (3)
C27	0.075 (3)	0.096 (4)	0.069 (3)	0.064 (3)	−0.008 (3)	0.000 (3)
C28	0.042 (2)	0.062 (2)	0.051 (2)	0.035 (2)	0.0038 (19)	0.005 (2)

### Geometric parameters (Å, °)

**Table:**

O1—N1	1.217 (6)	C11—C12	1.409 (6)
O2—N1	1.209 (7)	C12—C13	1.375 (6)
O3—C18	1.344 (5)	C12—H12	0.9300
O3—N3	1.410 (4)	C13—C14	1.390 (6)
O4—C28	1.221 (5)	C13—H13	0.9300
N1—C1	1.477 (6)	C14—C15	1.378 (5)
N2—C11	1.375 (5)	C14—H14	0.9300
N2—C7	1.454 (5)	C15—C16	1.496 (5)
N2—H2N	0.87 (3)	C16—C17	1.416 (5)
N3—C16	1.306 (5)	C17—C18	1.339 (5)
N4—C28	1.349 (5)	C17—H17	0.9300
N4—C25	1.457 (6)	C18—C19	1.477 (5)
N4—C9	1.472 (4)	C19—C20	1.378 (6)
C1—C2	1.353 (7)	C19—C24	1.379 (6)
C1—C6	1.370 (7)	C20—C21	1.390 (6)
C2—C3	1.362 (6)	C20—H20	0.9300
C2—H2	0.9300	C21—C22	1.370 (7)
C3—C4	1.381 (6)	C21—H21	0.9300
C3—H3	0.9300	C22—C23	1.346 (8)
C4—C5	1.389 (6)	C22—H22	0.9300
C4—C7	1.514 (6)	C23—C24	1.394 (7)
C5—C6	1.378 (8)	C23—H23	0.9300
C5—H5	0.9300	C24—H24	0.9300
C6—H6	0.9300	C25—C26	1.497 (7)
C7—C8	1.534 (5)	C25—H25A	0.9700
C7—H7	0.9800	C25—H25B	0.9700
C8—C9	1.527 (5)	C26—C27	1.486 (8)
C8—H8A	0.9700	C26—H26A	0.9700
C8—H8B	0.9700	C26—H26B	0.9700
C9—C10	1.523 (5)	C27—C28	1.515 (7)
C9—H9	0.9800	C27—H27A	0.9700
C10—C15	1.398 (5)	C27—H27B	0.9700
C10—C11	1.413 (5)		
			
C18—O3—N3	108.2 (3)	C12—C13—H13	119.9
O2—N1—O1	123.8 (5)	C14—C13—H13	119.9
O2—N1—C1	117.2 (5)	C15—C14—C13	119.8 (4)
O1—N1—C1	119.0 (5)	C15—C14—H14	120.1
C11—N2—C7	117.3 (3)	C13—C14—H14	120.1
C11—N2—H2N	117 (2)	C14—C15—C10	121.1 (3)
C7—N2—H2N	111.2 (19)	C14—C15—C16	119.9 (3)
C16—N3—O3	105.4 (3)	C10—C15—C16	118.9 (3)
C28—N4—C25	113.3 (3)	N3—C16—C17	111.6 (3)
C28—N4—C9	120.7 (3)	N3—C16—C15	118.0 (3)
C25—N4—C9	126.0 (3)	C17—C16—C15	130.4 (3)
C2—C1—C6	121.6 (4)	C18—C17—C16	104.4 (3)
C2—C1—N1	119.8 (5)	C18—C17—H17	127.8
C6—C1—N1	118.6 (5)	C16—C17—H17	127.8
C1—C2—C3	119.6 (5)	C17—C18—O3	110.3 (3)
C1—C2—H2	120.2	C17—C18—C19	133.0 (4)
C3—C2—H2	120.2	O3—C18—C19	116.6 (3)
C2—C3—C4	121.5 (4)	C20—C19—C24	119.5 (4)
C2—C3—H3	119.3	C20—C19—C18	119.9 (4)
C4—C3—H3	119.3	C24—C19—C18	120.7 (4)
C3—C4—C5	117.5 (4)	C19—C20—C21	120.5 (4)
C3—C4—C7	124.3 (4)	C19—C20—H20	119.7
C5—C4—C7	118.2 (4)	C21—C20—H20	119.7
C6—C5—C4	121.4 (5)	C22—C21—C20	119.2 (4)
C6—C5—H5	119.3	C22—C21—H21	120.4
C4—C5—H5	119.3	C20—C21—H21	120.4
C1—C6—C5	118.4 (5)	C23—C22—C21	120.6 (4)
C1—C6—H6	120.8	C23—C22—H22	119.7
C5—C6—H6	120.8	C21—C22—H22	119.7
N2—C7—C4	112.6 (3)	C22—C23—C24	121.0 (5)
N2—C7—C8	108.0 (3)	C22—C23—H23	119.5
C4—C7—C8	111.5 (4)	C24—C23—H23	119.5
N2—C7—H7	108.2	C19—C24—C23	119.1 (5)
C4—C7—H7	108.2	C19—C24—H24	120.4
C8—C7—H7	108.2	C23—C24—H24	120.4
C9—C8—C7	111.5 (3)	N4—C25—C26	105.1 (4)
C9—C8—H8A	109.3	N4—C25—H25A	110.7
C7—C8—H8A	109.3	C26—C25—H25A	110.7
C9—C8—H8B	109.3	N4—C25—H25B	110.7
C7—C8—H8B	109.3	C26—C25—H25B	110.7
H8A—C8—H8B	108.0	H25A—C25—H25B	108.8
N4—C9—C10	109.5 (3)	C27—C26—C25	107.0 (4)
N4—C9—C8	112.0 (3)	C27—C26—H26A	110.3
C10—C9—C8	113.2 (3)	C25—C26—H26A	110.3
N4—C9—H9	107.3	C27—C26—H26B	110.3
C10—C9—H9	107.3	C25—C26—H26B	110.3
C8—C9—H9	107.3	H26A—C26—H26B	108.6
C15—C10—C11	119.2 (3)	C26—C27—C28	105.7 (4)
C15—C10—C9	121.1 (3)	C26—C27—H27A	110.6
C11—C10—C9	119.6 (3)	C28—C27—H27A	110.6
N2—C11—C12	119.9 (3)	C26—C27—H27B	110.6
N2—C11—C10	121.7 (3)	C28—C27—H27B	110.6
C12—C11—C10	118.4 (3)	H27A—C27—H27B	108.7
C13—C12—C11	121.2 (3)	O4—C28—N4	125.3 (4)
C13—C12—H12	119.4	O4—C28—C27	126.7 (4)
C11—C12—H12	119.4	N4—C28—C27	108.0 (4)
C12—C13—C14	120.2 (3)		
			
C18—O3—N3—C16	−0.6 (4)	C12—C13—C14—C15	0.2 (6)
O2—N1—C1—C2	−174.9 (7)	C13—C14—C15—C10	−4.0 (6)
O1—N1—C1—C2	6.3 (8)	C13—C14—C15—C16	174.8 (4)
O2—N1—C1—C6	5.8 (9)	C11—C10—C15—C14	4.8 (5)
O1—N1—C1—C6	−173.0 (6)	C9—C10—C15—C14	−173.0 (3)
C6—C1—C2—C3	0.9 (8)	C11—C10—C15—C16	−174.0 (3)
N1—C1—C2—C3	−178.4 (5)	C9—C10—C15—C16	8.2 (5)
C1—C2—C3—C4	0.1 (8)	O3—N3—C16—C17	1.5 (4)
C2—C3—C4—C5	−1.0 (8)	O3—N3—C16—C15	−177.6 (3)
C2—C3—C4—C7	177.5 (5)	C14—C15—C16—N3	−103.9 (4)
C3—C4—C5—C6	1.1 (10)	C10—C15—C16—N3	74.9 (4)
C7—C4—C5—C6	−177.5 (6)	C14—C15—C16—C17	77.0 (5)
C2—C1—C6—C5	−0.8 (10)	C10—C15—C16—C17	−104.2 (5)
N1—C1—C6—C5	178.5 (6)	N3—C16—C17—C18	−1.9 (4)
C4—C5—C6—C1	−0.2 (11)	C15—C16—C17—C18	177.2 (4)
C11—N2—C7—C4	−177.3 (4)	C16—C17—C18—O3	1.4 (4)
C11—N2—C7—C8	−53.8 (5)	C16—C17—C18—C19	−176.3 (4)
C3—C4—C7—N2	29.5 (6)	N3—O3—C18—C17	−0.6 (4)
C5—C4—C7—N2	−152.0 (5)	N3—O3—C18—C19	177.6 (3)
C3—C4—C7—C8	−92.1 (5)	C17—C18—C19—C20	4.0 (6)
C5—C4—C7—C8	86.4 (6)	O3—C18—C19—C20	−173.6 (3)
N2—C7—C8—C9	59.0 (4)	C17—C18—C19—C24	−175.8 (5)
C4—C7—C8—C9	−176.8 (3)	O3—C18—C19—C24	6.6 (6)
C28—N4—C9—C10	−133.2 (3)	C24—C19—C20—C21	0.4 (6)
C25—N4—C9—C10	44.6 (5)	C18—C19—C20—C21	−179.4 (4)
C28—N4—C9—C8	100.4 (4)	C19—C20—C21—C22	0.6 (6)
C25—N4—C9—C8	−81.8 (5)	C20—C21—C22—C23	−1.7 (8)
C7—C8—C9—N4	85.5 (4)	C21—C22—C23—C24	1.9 (9)
C7—C8—C9—C10	−38.9 (4)	C20—C19—C24—C23	−0.2 (8)
N4—C9—C10—C15	63.8 (4)	C18—C19—C24—C23	179.5 (5)
C8—C9—C10—C15	−170.5 (3)	C22—C23—C24—C19	−0.9 (9)
N4—C9—C10—C11	−114.0 (4)	C28—N4—C25—C26	4.4 (6)
C8—C9—C10—C11	11.8 (5)	C9—N4—C25—C26	−173.5 (4)
C7—N2—C11—C12	−153.3 (4)	N4—C25—C26—C27	−8.6 (7)
C7—N2—C11—C10	27.5 (6)	C25—C26—C27—C28	9.5 (7)
C15—C10—C11—N2	177.3 (3)	C25—N4—C28—O4	−178.7 (4)
C9—C10—C11—N2	−4.9 (5)	C9—N4—C28—O4	−0.7 (6)
C15—C10—C11—C12	−1.9 (5)	C25—N4—C28—C27	1.6 (5)
C9—C10—C11—C12	175.9 (3)	C9—N4—C28—C27	179.6 (4)
N2—C11—C12—C13	179.0 (4)	C26—C27—C28—O4	173.3 (5)
C10—C11—C12—C13	−1.8 (6)	C26—C27—C28—N4	−7.0 (6)
C11—C12—C13—C14	2.7 (6)		

### Hydrogen-bond geometry (Å, °)

**Table:**

*D*—H···*A*	*D*—H	H···*A*	*D*···*A*	*D*—H···*A*
N2—H2N···O4^i^	0.87 (3)	1.99 (3)	2.859 (6)	180 (5)

Symmetry codes: (i) *y*, −*x*+*y*, *z*−1/6.
